# Developing an interprofessional decision support tool for diabetic foot ulcers management in primary care within the family medicine group model: a Delphi study in Canada

**DOI:** 10.1186/s12875-024-02387-4

**Published:** 2024-04-20

**Authors:** Magali Brousseau-Foley, Virginie Blanchette, Julie Houle, François Trudeau

**Affiliations:** 1 Department of Human Kinetics, Université du Québec à Trois-Rivières, Boul. Des Forges, Trois-Rivières, Québec, 3351G8Z 4M3 Canada; 2 Centre Intégré Universitaire de Santé et de Services Sociaux de la Mauricie et du Centre-du-Québec (CIUSSS-MCQ) affiliated to Université de Montréal, Department of Family and Emergency Medicine, Faculty of Medicine, 731 Rue Ste-Julie, Trois-Rivières, Québec, G9A 1Y1 Canada; 3 VITAM - Research Centre on Sustainable Health, 2480 Chemin de la Canardière, Québec, QC G1J 2G1 Canada; 4 Department of Nursing, Université du Québec à Trois-Rivières, 3351, Boul. Des Forges, Trois-Rivières, Québec, G8Z 4M3 Canada

**Keywords:** Diabetic foot ulcer, Decision support tool, Delphi protocol, Primary care, Family medicine groups, Interdisciplinarity, Care coordination

## Abstract

**Background:**

Primary care professionals encounter difficulties coordinating the continuum of care between primary care providers and second-line specialists and adhere to practice guidelines pertaining to diabetic foot ulcers management. Family medicine groups are providing primary care services aimed to improve access, interdisciplinary care, coordination and quality of health services, and reduce emergency department visits. Most professionals working in family medicine groups are primary care physicians and registered nurses. The aim of this study was to develop and validate an interprofessional decision support tool to guide the management of diabetic foot ulcers for primary care professionals working within the family medicine group model.

**Methods:**

A one-page decision tool developed by the research team was validated by an expert panel using a three-round Delphi protocol held between December 2019 and August 2021. The tool includes 43 individual actions and a care pathway from initial presentation to secondary prevention. Data collection was realized with both paper and electronic questionnaires, and answers were compiled in an electronic spreadsheet. Data was analyzed with use of descriptive statistics, and consensus for each item was defined as ≥ 80% agreement.

**Results:**

Experts from 12 pre-identified professions of the diabetic foot ulcer interdisciplinary care team were included, 39 participants out of the 59 invited to first round (66.1%), 34 out of 39 for second (87.2%) and 22 out of 34 for third (64.7%) rounds. All items included in the final version of the decision support tool reached consensus and were deemed clear, relevant and feasible. One or more professionals were identified to be responsible for every action to be taken.

**Conclusions:**

This study provided a comprehensive decision support tool to guide primary care professionals in the management of diabetic foot ulcers. Implementation and evaluation in the clinical setting will need to be undertaken in the future.

**Supplementary Information:**

The online version contains supplementary material available at 10.1186/s12875-024-02387-4.

## Introduction

Diabetes mellitus prevalence has been constantly on the rise for over two decades. Worldwide, 536.6 million people were living with diabetes in 2021 [1]. In Canada, this represents 10% of the population in 2022 [2]. Lifetime risk of suffering from a diabetes-related foot ulcer (DFU) is estimated at 34%, with a 20% risk of limb amputation [3]. A DFU is defined as “a foot ulcer in a person with current or previously diagnosed diabetes mellitus, and usually accompanied by peripheral neuropathy and/or peripheral artery disease in the lower extremity” [4]. Adherence to a comprehensive evidence-based coordinated treatment regimen provided by an interdisciplinary care team decreases the need for major amputations and improves healing rates [3, 5, 6]. However, current clinical practices in primary care, specifically within the family medicine group (FMG) model in the province of Quebec, Canada, have difficulties to adhere to DFUs management guidelines, plus coordinating the continuum of care between primary care professionals and second-line specialists. An audit previously conducted by our research team highlighted some issues that led people with diabetes to be admitted to our regional hospital because of a DFU or a DFU complication as the main admission diagnosis [7]. The following discrepancies from current best practice recommendations were identified: absence of a DFU team resulting in poor coordination of care, silo work from health professionals, inefficient communication between stakeholders, lack of knowledge about the scope of practice of other professions relevant to DFU care, insufficient wound care training and inability to prioritize concurrent health needs in this complex population. Therefore, there is a failure to provide their patients with the best possible chance for healing DFUs and avoiding amputations [8–10]. FMGs provide most of the primary care services to Quebec’s population since their establishment in 2002 as a means to improve access, interdisciplinary care, coordination and quality of health services, and reduce emergency department visits [11, 12]. FMGs professionals are mostly primary care physicians (PCPs) supported by registered nurses (RN) usually in a 4 to 6 PCPs to 1 RN ratio based on number of people enrolled in the FMG. It encourages PCPs to be available in priority, if not exclusively, to people enrolled in his or her FMG. The model was developed so RN could alleviate PCPs workload by taking care of minor ailments not requiring physician expertise [13]. Other healthcare professionals (physiotherapists, psychologist, social workers, etc.) are also sometimes present. Moreover, in exchange for its staff and material resources being partially financed by the public health system, the FMG has the obligation to offer medical services in the evenings and weekends. The FMG model was therefore mostly developed to provide increased access and continuity of care in the mean of extended practice hours and added professionals, rather than true interdisciplinary care, which is more dependent on each clinic local organization and available staff [14]. Despite its specificities, the FMG model in Quebec, Canada, can been seen as a variation of other team-based primary care models elsewhere in the world where professionals offer longitudinal continuous general healthcare services [12]. In the absence of dedicated wound clinics, DFU assessment and management often falls under the responsibility of FMGs professionals, whom then frequently refer patients to state-financed healthcare community services or podiatric private practices when patients have private insurances covering these services.

The need for a decision support tool to guide DFUs’ management has already been expressed in various clinical settings [15–18] but was never addressed specifically for the Quebec’s FMG model, where only a few clinical settings have access to specialized wounds clinics [8, 19, 20]. Practice guidelines originating from national [21–23] and international interest groups [6, 24] are widely available but often represent documents not practical in clinical setting. Additionally, implementation of guidelines can be challenging as their recommendations can be heterogeneous [25]. Decision support tools developed for other complex medical problems managed in primary care proved to help with achieving better quality and more coherent care [26, 27]. We therefore advocate that a decision support tool for DFU management in primary care could similarly improve outcomes in this population. A Delphi protocol was chosen as the most effective method to develop and validate the decision support tool. A Delphi protocol is an iterative, structured process, widely used in multiple disciplines, namely in health sciences, to obtain a consensus from answers to questionnaires based on anonymous opinions of a group of participants selected because of their personal expertise on a topic under study [28]. A Delphi protocol have previously been used to develop and validate decision aid tools for primary care professionals [29, 30] and DFU management [31, 32]. The purpose of this study is to develop and validate a comprehensive decision support tool to guide the management of DFUs diagnosed by professionals within FMGs. This validation process is necessary in order to ensure that the tool is relevant, clear and feasible before its implementation.

## Methods

### Decision support tool development

The interdisciplinary research team (MBF, VB, JH) and collaborators (a PCP, a RN and a podiatrist) developed a one-page decision tool to help FMGs’ professionals manage DFUs based on a rapid review of current literature including practice guidelines and related evidence-based literature [33] during the fall of 2019. Identified actions to be taken in the management of DFUs were classified according to the severity (uncomplicated vs complicated) and divided into professional roles to reflect what was available from the literature as well as professional practices in the province of Quebec.

### Decision support tool validation

The initial version (43 items) of the decision support tool was validated through a modified Delphi protocol. This Delphi validation was led and reported according to the Guidance on Conducting and REporting DElphi Studies (CREDES) [34]. Because of the complexity of producing a structured, comprehensive and multidisciplinary tool, it was decided to diverge from the classical Delphi protocol which usually presents participants with open-ended questions in order to generate qualitative data that will become the items to be evaluated for consensus in subsequent rounds, hence the modified Delphi protocol [28]. The alternative pathways represented in the tool reflect well the adjustable composition of the ideal team [23, 35]. Also, because of the facilitator’s role of filtering participants’ answers and providing controlled feedback, the final version of the tool cannot be totally exempt from the subjective interpretation of the authors on the matter [28]. A maximum number of three rounds to achieve consensus was predetermined in accordance with scientific evidence [28].

**Fig. 1:**
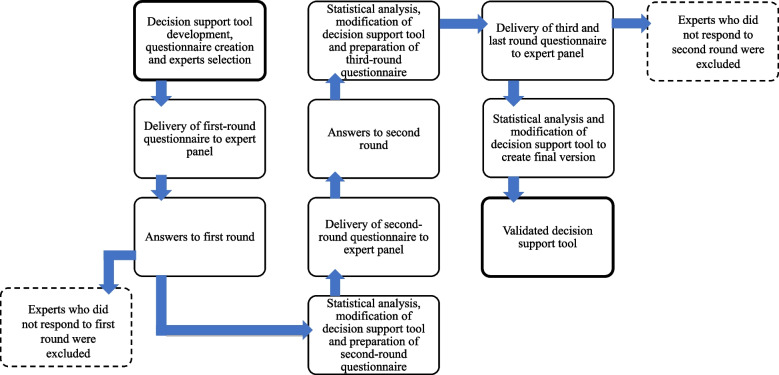
Flow chart illustrating the stages of the modified Delphi protocol

Figure 1 illustrates the modified Delphi protocol. Expert panel’s recommendations were collected for the first round from December 2019 to May 2020 using a three-part paper questionnaire and a one-page decision support tool in its initial version. Questionnaires developed by the research team for all three rounds are available in translated English language versions in Additional file 1. The first part included 13 questions about participants sociodemographic and professional characteristics. The second part included 43 questions corresponding to each item of the decision support tool. For each item, the expert had to evaluate four criteria: the item is clear, relevant, feasible, and which healthcare professional should be responsible for it. The following definitions [36] were provided to guide participants:

- Clarity means that the wording to describe the item is easily understandable;
- Relevance indicates that the item is in relation to the matter at hand;
- Feasibility means that one is capable of doing or of carrying out the action; and
- Responsibility is liability for an action (based on the professional scope of practice, competencies and availability in the healthcare system organization).

The third part of the questionnaire asked three open-ended questions: 1) regarding additional items or supplementary resources that should have been included in the decision support tool, 2) if the graphic layout and organization of the tool was user-friendly and straightforward, and 3) if the participant had any additional comments. The time required to complete the first-round questionnaire was estimated at 60 to 90 min.

The second and third Delphi rounds were conducted through an online questionnaire using the Université du Québec à Trois-Rivières online questionnaire tool from March to May 2021 and from July to September 2021 respectively. The time required to complete questionnaires was approximately 15 min for the second round and approximately 5 min for the third round. The format was similar to the second part of the first-round questionnaire. A new section of the questionnaire allowed participants to determine which supplementary resources proposed in the first round should be included into the decision support tool. All experts from the first round were invited to participate. Items from the first round that reached consensus were removed. Controlled feedback regarding the previous round statistical aggregation of experts’ answers was provided both with an updated version of the decision support tool and through the questions developed from first-round items that did not meet consensus. The second-round questionnaire comprised a total of 34 questions.

For the third round of the Delphi questionnaire, all experts from the second round were invited to participate in the third round. Feedback regarding the previous round statistical aggregation of experts’ answers was provided both with an updated version of the decision support tool and through the questions developed from second-round items that did not meet consensus. The third-round questionnaire comprised a total of 4 questions.

### Participants

As the projected decision support tool users are primary care professionals, an expert panel to validate its content and structure needed to include both content experts but also professionals with wound care management expertise representative of most professionals working in this environment. We aimed at inviting for the first round’s questionnaire five professionals from each 12 predetermined areas of expertise: 1) PCPs, 2) RNs, 3) podiatrists, 4) RNs specialized in wounds, 5) physiatrists, 6) occupational therapists, 7) physiotherapists, 8) orthotists, 9) infectious disease or internal medicine physicians, 10) vascular surgeons, 11) orthopedic surgeons, and 12) wound care researchers for a total of about 60 participants. An expert panel’s member had to individually and anonymously share their opinion in the questionnaires. All experts needed to have knowledge or clinical experience with the management of DFU in the province of Quebec, Canada. They had to be competent in French as this is the language in which the tool was developed, French being the official language of this province. Participants identified by the authors in their network of contacts were individually solicited to participate. All received an information letter and consent form to be signed. Delphi rounds took place between December 2019 and August 2021. The sample size was determined according to what is recommended for this study design [28] and based on the expected initial response rate and attrition rate throughout sequential Delphi rounds.

### Ethics approval

This research project was performed in accordance with the declaration of Helsinki and received ethical approbation from the Centre intégré universitaire de santé et de services sociaux de la Mauricie-et-du-Centre-du-Québec ethical board CÉRM-2019–002. Written informed consent was obtained from all subjects.

### Statistical analysis

All answers were entered into an Excel spreadsheet (Microsoft, version 16.16.7) for quantitative and qualitative analysis. Consensus threshold was defined as 80% agreement for each item. Clarity was noted as 0 (unclear) or 1 (clear). All items that did not reach 80% agreement for clarity were submitted with alternative wording in the next round. Relevance was evaluated with a four-point Likert scale (1 being of low relevance and 4 high relevance). For each item, values of three and four were considered as agreement. Feasibility was evaluated with a five-point Likert scale (1 being of low feasibility and 5 high feasibility). For each item, values four and five were considered as agreement. A relative frequency of 80% or higher of values three and four for an item was considered to have reached agreement consensus and was excluded from the next round. The choice between a four-point and a five-point Likert scale was based on the nature of the data to collect. Regarding relevance, because items were identified from evidence-based literature, a higher consensus was expected for the relevance criterion and it was chosen to avoid the possibility of a neutral response in order to force experts to take a stance. On the other hand, as resources are often limited within the healthcare system and vary in different geographical locations, neutral responses on a five-point scale was considered unfeasible and therefore excluded the item, as the tool had to propose actions achievable for most professionals no matter the care setting. For the responsibility criterion, participants had to choose from a predetermined list of professionals (PCP, RN, podiatrist, RN specialized in wound care, rehabilitation team, infectious disease specialist, vascular surgeon and orthopedic surgeon) or other and specify the professional title. The relative frequency in percentage of answers was calculated for each item. A choice of professional that reached a relative frequency of 20% and higher for an item was retained. It was therefore possible to have more than one professional identified as responsible for an item. In the absence of a clear consensus, all choices of professional individually and in all possible combinations were submitted in the second round. All open-ended answers were manually organized by subject and theme in the same spreadsheet. All additional resources to be added to the decision support tool suggested by participants more than three times in answer to open-ended questions were listed to be evaluated in the second round. In the second round, for each item and criterion, consensus threshold was calculated in the same manner as in the first round. Supplementary resources were evaluated using four-point Likert scales (1 being of low relevance and 4 high relevance of the proposed additional resource). Values of three and four were considered as agreement. A relative frequency of 80% or higher of values three and four for an item was considered to have reached agreement threshold. Supplementary resources that did not reach consensus were excluded. For the third Delphi round, a simple majority (50% and more agreement) was required to achieve consensus on each item and criterion.

### Final version of decision aid tool

As the initial version of the decision aid tool was developed based on literature available in 2019, once data collection and analysis were completed, the research team verified if any significant changes appeared in national and international guidelines and best practice documents between 2019 and 2023 that would require to add to, remove or modify any items included in the tool. As for the additional resources to appear at the back of the tool selected by the expert panel, the hyperlinks and resources provided were chosen by the research team in 2023, prioritizing national resources when available and if not, resources from international organizations.

## Results

### Delphi first round

Fifty-nine experts were invited to participate in the study. A total of 39 participants (66.1% response rate) returned the completed first-round questionnaire (mean age = 40.9 years old; SD = 10.21 years). All professional titles were represented by at least two participants except for occupational therapist which was not present. The first-round panel was composed mostly of PCPs and RNs (Table 1). A majority of experts were working in clinical settings (Table 2).

**Table 1:** Professional title of participants in the expert panel

Professional title	Number of participants	Percentage of expert panel (%)
Primary care physicians^a^	8	20.51
Registered nurses	7	17.95
Podiatrists	5	12.82
Registered nurses specialized in wound care	5	12.82
Infectious disease and internal medicine physicians	4	10.26
Rehabilitation professionals (physiatrists, occupational therapists, physiotherapists, orthotists)	3	7.69
Vascular surgeons	3	7.69
Orthopedic surgeons	2	5.13
Wound care researchers	1	2.56
Other^a^	1	2.56
Total	39	100.00

^a^Emergency physician also involved in wound care research

**Table 2:** Distribution of participants according to their main work setting

Work setting	Number of participants	Percentage of expert panel (%)
Hospital	20	51.28
FMGs	10	25.64
Wound care center	2	5.13
Other type of clinical setting (private practice)	4	10.26
Non-clinical	3	7.69
Total	39	100.00

*FMGs* family medicine groups.

Participants were mostly women (64%). Participants accumulated 14.7 ± 9.2 (M ± SD) years of professional experience in their field and a mean of 8.3 ± 8.5 years specifically in wound care. Agreement obtained for all items and criteria for each Delphi round is detailed in Table 3.

**Table 3:** Agreement reached in the three Delphi rounds for the four criteria evaluated for each item

Criteria	Clarity	Relevance	Feasibility	Responsibility
First round				
	42/43 (97.7%)	41/43 (95.3%)	38/43 (88.4%)	40/43 (93.0%)
				3 items yielding 13 combinations of professionals
Second round				
	0/1 (0%)	1/2 (50%)	4/5 (80%)	12/13 (92.3%)
	Choice of 2 propositions			
Third round				
	1 proposition reached 66% agreement	1 remaining item reached 81.8% agreement	1 remaining item reached 77.3% agreement	1 remaining item reached 54.6% agreement

For responsibility, three items were shared between more than one professional, resulting in 13 possible individual professionals or combinations of professionals. Out of the 13 supplementary resources to be added to the decision support tool, nine reached consensus. The decision support tool was adjusted based on these results by the authors and graphic designer.

### Delphi second round

Out of the 39 experts invited to the second round, a total of 34 participants (87.2% response rate) completed the second-round questionnaire. All professional titles were represented by at least two participants except for physiotherapist and occupational therapist. A majority of PCPs, RNs and RNs specialized in wounds made for 66.6% (26 participants) of the expert panel. Based on agreement, the decision support tool was again adjusted based on these results by the authors and graphic designer.

### Delphi third round

**Fig. 2:**
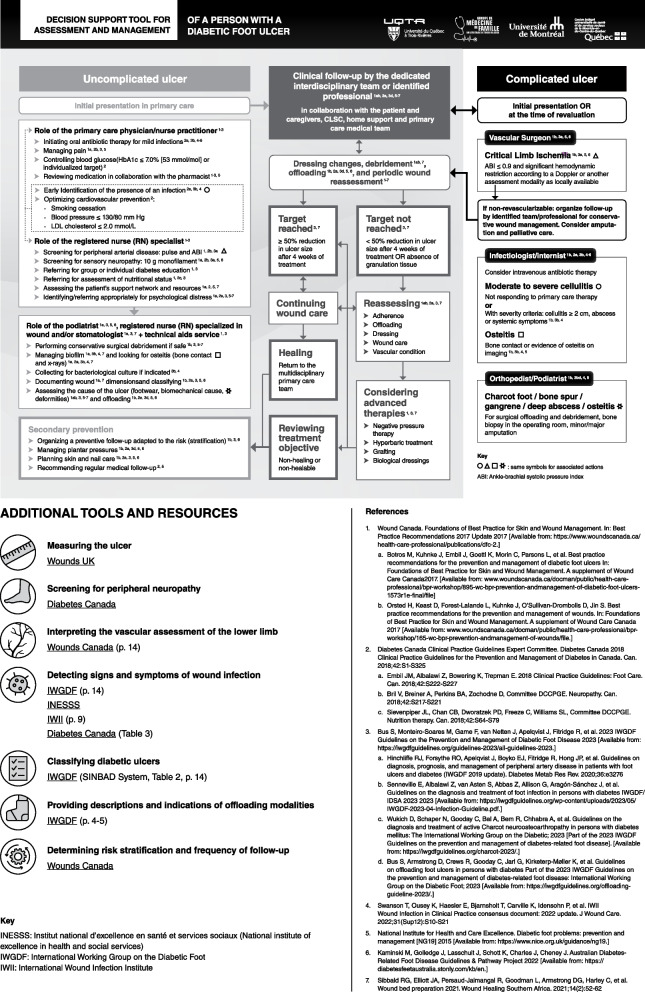
Final version of the front page of the decision support tool (translated to English)

Out of the 34 experts invited to the second round, a total of 22 participants (64.7% response rate) completed the third-round questionnaire. All professional titles were represented by at least one participant (infectious disease physician and orthotist) or more, except for physiotherapist, occupational therapist and orthopedic surgeon. A majority of PCPs, RNs and RNs specialized in wounds made for 63.6% (14 participants) of the expert panel. The decision support tool was adjusted for its final version based on the attained results, which was translated to English (Fig. 2). The complete final version of the tool in color and translated to English is available in Additional file 2. The original French version in available in Additional file 3.

The front page of the tool is divided into three columns of different colors, from left to right green, orange and red, respectively representing actions to be taken for uncomplicated ulcers, wound care and complicated ulcers. The green column includes actions under the responsibility of primary care professionals (PCPs, RNs, and podiatrists if available). The orange column shows actions that may be realized by first or second-line professionals or by professionals working in community or private settings dependent on local care organizations. The red column lists actions and specifies reasons for referral to second-line specialists. Arrows indicate the direction of the pathway at different steps along the continuum of care, and symbols highlight important actions where primary care professionals might consider a referral to a second-line specialist. An updated rapid-review of the literature available in 2023 after data analysis was completed and did not compel any change in the items validated by the expert panel. The back of the tool provides up-to-date references and hyperlinks for additional resources to further inform and educate professionals to help them achieve evidence-based practices.

## Discussion

The purpose of this study was to develop and validate a comprehensive decision support tool to guide the assessment and management of DFUs in primary care. The tool was produced and validated using a Delphi protocol by an expert panel including professionals susceptible to compose an ideal interdisciplinary specialized wound care team. The tool targets primary care professionals in order to guide them in delivering coordinated care following up to date practice guidelines to people with DFU. It also serves to improve communications and trajectories between primary care professionals and second-line specialists. It is not intended to be used for screening and stratification of the at-risk diabetic foot in primary care as a recent validated tool is already available [37]. As any decision aid tool, it should not take precedent over health professionals’ clinical judgment.

It is demonstrated that timely and coordinated interventions for people with DFU with a specialized team involved in integrated prevention and care obtain better outcomes, namely improve DFU healing rates [35], reduce major amputations [38, 39], and lower health care costs [40, 41]. However, DFU specialized teams are not available and integrated care is absent in the majority of Quebec and Canadian regions and there is heterogeneity in the resources available locally [42]. To our knowledge, this is the first study in Canada to develop and validate a decision support tool intended for FMG’s professionals, mostly PCPs and RNs, to be used as a care pathway and checklist of actions to be completed when taking care of people with a DFU. A recent study implemented an acute care DFU pathway for people requiring hospitalization, reducing length of stay and costs [18]. Yet, the interest of a tool to be used in primary care is to decrease the necessity for people with DFU to visit the emergency department and to be admitted to hospital. This is known to reduce costs [43] and morbidity [44] associated with hospital stays. We postulate that our tool could improve primary care professionals’ capacity to manage DFU and consequently could enhance outpatient care. This also aligns with the primary purpose that led to the development of FMGs, namely to provide continuity of care outside of the hospital setting [13]. FMGs’ teams usually know their enrollees well and are easily accessible, especially for those with chronic diseases such as diabetes. In that perspective, the patient’s primary care team is often best placed to coordinate the complex needs arising with the occurrence of a DFU. Unfortunately, many obstacles still exist in the healthcare system organization that sometimes makes it easier for professionals and patients to manage DFUs in a hospital setting.

The comments formulated by the expert panel members were not formally analyzed but were considered by the research team when adjusting the tool after each Delphi round. One main theme that was recurrent is the lack of equipment, poor access to the hospital technical platform and limited availability of professionals outside the FMG staff. This was especially true regarding the vascular evaluation that needs to be performed on initial presentation of a person with a DFU. Having access to simple equipment such as a portable Doppler with the proper probe was reported to be challenging for many experts. Even in a hospital setting, well-equipped vascular labs appeared to be as rare as dedicated interprofessional wound care teams. This explains why toe pressure or toe pressure index was not included in the tool. Also, long delays to obtain arterial Doppler or angiography through a radiology department was perceived to have a negative impact on DFU care quality. Current practice for most experts was therefore to consult with a vascular surgeon when vascular status was uncertain, and the tool was made to reflect this. Health promotion interventions such as nutritional evaluation and diabetic education were also identified as actions for which there were insufficient resources available, even though many experts highlighted the importance of patients’ empowerment and education to self-management of disease. In this instance, it was mostly due to time constraints rather than equipment or expertise as FMG professionals were deemed capable of providing this service, but the difficulty resided in time limitation secondary to an already excessive work load. Access to nutritionists and diabetes educators was also challenging for patients that depend solely on resources available through publicly funded healthcare. Similarly, lack of access to podiatrists, which is not covered by the public healthcare system in Quebec, was judged to have a negative impact on DFU management as they were considered by most experts to be the best professionals to provide wound debridement and decide on offloading modality. When a podiatrist was not implicated, many experts felt debridement and offloading were not done or were inadequately done. Finally, a very significant obstacle that was pointed out by numerous members of the expert panel is that most offloading modalities have to be paid out of pocket by patients, and for a large proportion, this financial burden is too much, transforming healable DFUs into maintenance wounds.

Access to limb preservation interventions prior to hospitalization is known to be heterogenous as shown in Ontario (Canada) and seems particularly inadequate in regions distant from major medical centers [45]. Providing PCPs with standardized criteria for referral to second line specialists before amputation becomes unavoidable could improve relevance and timing of consultations with vascular and orthopedic surgeons. It could also improve communication and facilitates collaboration between PCPs and tertiary center consultants through telemedicine when local resources are not available. Telemedicine has demonstrated benefits for the management of many complex diseases, including DFU [46]. Because the decision support tool is intended for primary care professionals, the same team of professionals that will continue to provide comprehensive healthcare after the DFU episode, their interventions might have a beneficial impact lowering DFU recurrence rate and improve limb preservation, as about 80% of amputations are preventable [47]. Such a tool as also the potential to serve as a basis to discuss basic prevention and health promotion interventions between health professionals and patients as it provides an overview of all that require consideration when caring for people with DFU. It might be of interest that future studies aim at transforming this tool into a knowledge transfer tool both for health professionals, their patients and caregivers.

It is needed to mention that the rapid-review that was conducted to develop the initial version of the decision aid tool submitted during the initial round of the Delphi validation was completed in 2019, and the three rounds of questionnaires took place from 2019 to 2021. However, no specific references were provided to the expert panel during the Delphi validation, only a list of items to be evaluated. Also, most items stated general recommendations regarding DFU evaluation and management which are unlikely to evolve with time so significantly as to become obsolete. Therefore, once data collection and analysis were completed, the research team verified if any changes appeared in national and international guidelines and best practice documents between 2019 and 2023 that would require to add to, remove or modify any items included in the tool. As there were no significant changes, the reference list was updated, and the tool remained the same. As for the additional resources provided at the back of the tool, the expert panel only provided a list of themes pertaining to DFU management rather than specific resources, therefore, the hyperlinks and resources selected were chosen by the research team once the Delphi process was completed, prioritizing national resources when available and if not, resources from international organizations. The English (Fig. 2 and Additional File 2) and French (Additional File 3) versions are different as in the original French version of the tool, French language resources were prioritized over English language resources when available and up to date.

### Limitations

Some of the limitations inherent to the study design is the selection process of the expert panel, which could introduce a selection bias. However, we consider that our sample was representative in composition and proportions of both the main users (primary care professionals and second line specialists) and available resources within the local healthcare system organization. Even though only 25.64% of the first-round expert panel members currently worked within a FMG as their main occupation, many worked within a FMG as a secondary occupation or had worked in a FMG in the past. Moreover, 38.46% of experts were PCPs or RNs, the two mandatory professions composing FMGs. One member of the research team (MBF) who participated in all steps of this study is also a PCP working in a FMG. The response rate and presence of most professional titles in each round were acceptable for this study design [48, 49], especially considering that professionals’ availability was more challenging due to the ongoing COVID-19 pandemic at the time of data collection. Also, even though the FMG model is specific to the province of Quebec, Canada, it is merely a variation of many other primary care models elsewhere in Canada and worldwide as it is a team-based approach in which primary care professionals (mostly PCPs and RNs supported by other first line professionals) provide longitudinal continuous general healthcare services [12]. Therefore, we think that the validated tool could easily be adapted and implemented in other similar settings. Another limit to our study is that no patient was part of the research team. This means that even though the decision support tool was validated by health professionals, it might not be well adapted to DFU patients’ needs. This perspective could however be addressed in the future in a patient-oriented research project, and the tool adapted to the findings of such a study.

## Conclusions

This study describes the development process and validation of a decision support tool to guide primary care professionals manage DFUs based on current practice guidelines through a modified Delphi protocol. Our tool is all at once a checklist of actions that need to be taken by different professionals, a care pathway and a quick read reference for professionals to be informed about recommended treatments and expected outcomes at different steps along the continuum of care. We believe that it has the potential to contribute to standardizing and to optimizing the provision of care for people with DFUs in primary care. The implementation and evaluation of the tool in the clinical setting will be undertaken as a next step to determine if its use impacts on users’ satisfaction both for professionals and patients, as well as on clinical outcomes.

### Supplementary Information

**Additional file 1. **Questionnaires developed by the research team for all three Delphi rounds in translated English language versions.

**Additional file 2. **Final version of the decision support tool in colors translated to English.

**Additional file 3. **Original French version of the final version of the decision support tool.

## Data Availability

The datasets used and/or analysed during the current study are available from the corresponding author on reasonable request.

## Abbreviations

DFU: Diabetic foot ulcer
FMG: Family medicine group
RN: Registered nurse
PCP: Primary care physician
