# Supplementary material for: Developing an interprofessional decision support tool for diabetic foot ulcers management in primary care within the family medicine group model: a Delphi study in Canada
Source: BMC Prim Care. 2024 Apr 20;25:123. doi: 10.1186/s12875-024-02387-4 (PMC11031884; doi:10.1186/s12875-024-02387-4)
Supplement: Supplementary file 1 — Additional file 1. Questionnaires developed by the research team for all three Delphi rounds in translated English language versions. [file 12875_2024_2387_MOESM1_ESM.pdf]

Delphi first round questionnaire
(translated to English language)

Validation Questionnaire for the Decision Support Tool for the Evaluation and Management of a Diabetic Patient with Foot Ulcer

It is recognized that the management of diabetic foot ulcers (DFUs)* is more optimal when carried out by a multidisciplinary team. Unfortunately, few clinical settings in Quebec currently have access to this type of organization. It therefore appears relevant to better structure the efforts of the existing workforce in order to maximize the care that can be provided at the primary care level and to also improve the relevance and prioritization of cases requiring specialized medical involvement.

We would like to thank you for your participation in our efforts to develop a decision support tool to guide frontline healthcare providers in the evaluation and management of DFUs (Diabetic Foot Ulcers). This questionnaire aims to validate the elements that should be included in the evaluation and management of DFUs at the primary care level, the roles and interventions assigned to various healthcare professionals involved in clinical care, and the order in which these actions should be carried out. Responding to the questionnaire should take approximately 30 minutes of your time. We encourage you to provide your feedback and let us know if there are any additional elements you consider relevant to be added to the tool. If you agree, you may be invited to respond to an additional follow-up questionnaire related to any changes made to the tool based on the feedback received.

With the help of this questionnaire, we seek your opinion on the following:

- The clarity of the proposed statements and the understanding of the elements related to the evaluation or management of DFUs. À
- The relevance of considering each of the roles and elements mentioned for the intervention with a person with a DFU.
- The critical elements of evaluation and management, meaning actions that, if omitted, carry a high potential for complications.
- The assignment of responsibility for roles and assessments in the management to the appropriate healthcare provider.
- The feasibility, accessibility, or realism of the elements, i.e., the ease with which this healthcare provider could realistically fulfill this role in practice.

Responses to the questionnaire are collected anonymously. You do not need to provide your name on this questionnaire. Alphanumeric codes known only to the research team will be used to ensure your anonymity and confidentiality. The responses you provide will not be associated with your employee record in any way. Your identity and that of your affiliated institution will not be disclosed in any potential publication. The collected data will be retained for a period of 5 years in a locked file at the Université du Québec à Trois-Rivières, accessible only to the principal investigator of the project. The data will be destroyed at the end of the specified period.

If you have any questions regarding this project, you are invited to contact the principal investigator using the following contact information.

The research team

* A diabetic foot ulcer represents any damage to the skin or underlying tissues below the level of the ankle in a person with diabetes.

PART 1 Respondent characteristics

Age: _______

Gender: □ Male □ Female

Discipline (check all that apply):
□ Primary Care Physician
□ Registered Nurse
□ Registered Nurse with certification and authorized to prescribe in wound care under the Regulation on certain professional activities that can be performed by a nurse
□ Podiatrist
□ Registered nurse specialized in wounds
□ Assistive Technology Service (specify profession): ________________________
□ Physiotherapist
□ Physiotherapist primarily working in wound care
□ Occupational Therapist
□ Infectious Disease Specialist
□ Vascular Surgeon
□ Orthopedic Surgeon
□ Other (specify): ________________________

Primary Practice Setting (choose one):
□ Family Medicine Group (FMG)
□ Other Medical Clinic (specify): ________________________
□ Hospital
□ Wound Care Center
□ Not in a clinic (specify): ________________________

Percentage of your activities in this setting: _________ %

Sociosanitary Region of Primary Practice Setting (choose one):
□ Bas-Saint-Laurent
□ Saguenay – Lac-St-Jean
□ Capitale-Nationale
□ Mauricie et Centre-du-Québec
□ Estrie
□ Montreal
□ Outaouais
□ Abitibi-Témiscamingue
□ Côte-Nord
□ Nord-du-Québec

□ Gaspésie-Îles-de-la-Madeleine
□ Chaudières-Appalaches
□ Laval
□ Lanaudière
□ Montérégie
□ Nunavik
□ Terres-Cries-de-la-Baie-James

Secondary practice setting, if applicable (choose one):
□ Family Medicine Group (FMG)
□ Other Medical Clinic (specify): ________________________
□ Hospital
□ Wound Care Center
□ Not in a clinic (specify): ________________________

Percentage of your activities in this setting: _________ %

Highest level of education obtained (choose one):
□ Undergraduate Bachelor's Degree
□ Professional Doctorate (e.g., Doctor of Medicine)
□ Graduate Master's Degree in Research
□ Graduate Professional Master's Degree (e.g., Nurse Practitioner)
□ Doctoral Degree (Ph.D.)
□ Postdoctoral Research
Field of study: _________________________________

Total years of clinical practice, if applicable: ________

Total years of clinical practice in wound care, if applicable: ________

If applicable, continuing education related to wound care (e.g., Graduate Diploma, microprogram, other accredited or non-accredited courses, etc.). Please name these courses:
• ____
• ____
• ____

On a scale of 1 to 5, with 1 representing no personal interest and 5 representing extremely high personal interest in the subject of DFUs, where do you place yourself?
□ 1 □ 2 □ 3 □ 4 □ 5

On a scale of 1 to 5, with 1 representing no personal interest and 5 representing extremely high personal interest in participating in continuing education activities on the subject of DFUs, where do you place yourself?

□ 1 □ 2 □ 3 □ 4 □ 5

On a scale of 1 to 5, with 1 representing no sense of competence and 5 representing an extremely high sense of competence in mastering the latest guidelines for the management of DFUs, where do you place yourself?
□ 1 □ 2 □ 3 □ 4 □ 5

Average number of visits by patients with DFUs in which you participate per month:
□ 0-5
□ 6-15
□ 16-25
□ More than 25
□ N/A

Please complete Part 2 of the questionnaire before answering the following questions.

What other roles or elements for the evaluation or management of DFUs to guide frontline providers should be included in the proposed tool?

As you may have noticed, there are arrows and flags at certain strategic points in the decision support tool. What is your assessment of the organization and sequential logic of the proposed tool's use? Please comment.

Please provide any other comments regarding the evaluation and management of DFUs that have not been discussed elsewhere.

PART 2 Evaluation of decision support tool

Roles and Elements of Evaluation and Management	**Clarity**<br>Check if the description is clear or not<br>Unclear. Please provide comments	Clear	**Relevance**<br>Evaluate the relevance of each element for the assessment and management of a DFU from 1 (not relevant) to 4 (very relevant)	**Critical element**<br>Check if it is a critical element	**Responsibility**<br>Check a single healthcare provider who, in your opinion, should be responsible for this element	**Feasibility**<br>Evaluate the feasibility of each element for the healthcare provider in their clinical setting, from 1 (impossible to accomplish) to 5 (very easily achievable)	Comments

UNCOMPLICATED ULCER - INITIAL PRESENTATION IN PRIMARY CARE (Green Section)

1	Roles of primary care physician							
1a	Early identification of the presence of an infection			1 2 3 4		☐ Primary care physician<br>☐ Registered nurse<br>☐ Podiatrist<br>☐ Registered nurse specialized in wounds<br>☐ Assistive Technology Service ☐Infectious disease specialist<br>☐ Vascular surgeon<br>☐ Orthopedic surgeon<br>☐ Other, specify:	1 2 3 4 5	
1b	Initiate oral antibiotic therapy in the case of mild infection			1 2 3 4		☐ Primary care physician<br>☐ Registered nurse<br>☐ Podiatrist<br>☐ Registered nurse specialized in wounds<br>☐ Assistive Technology Service ☐Infectious disease specialist<br>☐ Vascular surgeon<br>☐ Orthopedic surgeon<br>☐ Other, specify:	1 2 3 4 5	

1c	Manage pain			1 2 3 4		☐ Primary care physician<br>☐ Registered nurse<br>☐ Podiatrist<br>☐ Registered nurse specialized in wounds<br>☐ Assistive Technology Service ☐Infectious disease specialist<br>☐ Vascular surgeon<br>☐ Orthopedic surgeon<br>☐ Other, specify:	1 2 3 4 5	
1d	Control blood glucose (HbA1c ≤ 7.0% or individualized target)			1 2 3 4		☐ Primary care physician<br>☐ Registered nurse<br>☐ Podiatrist<br>☐ Registered nurse specialized in wounds<br>☐ Assistive Technology Service ☐Infectious disease specialist<br>☐ Vascular surgeon<br>☐ Orthopedic surgeon<br>☐ Other, specify:	1 2 3 4 5	
1e	Review medication			1 2 3 4		☐ Primary care physician<br>☐ Registered nurse<br>☐ Podiatrist<br>☐ Registered nurse specialized in wounds<br>☐ Assistive Technology Service ☐Infectious disease specialist<br>☐ Vascular surgeon<br>☐ Orthopedic surgeon<br>☐ Other, specify:	1 2 3 4 5	
1f	Optimize cardiovascular prevention: Smoking cessation			1 2 3 4		☐ Primary care physician<br>☐ Registered nurse<br>☐ Podiatrist<br>☐ Registered nurse specialized in wounds<br>☐ Assistive Technology Service ☐Infectious disease specialist	1 2 3 4 5	

						☐ Vascular surgeon<br>☐ Orthopedic surgeon<br>☐ Other, specify:		
1g	Optimize cardiovascular prevention: Blood pressure ≤ 130/80 mm Hg			**1 2 3 4**		☐ Primary care physician<br>☐ Registered nurse<br>☐ Podiatrist<br>☐ Registered nurse specialized in wounds<br>☐ Assistive Technology Service ☐Infectious disease specialist<br>☐ Vascular surgeon<br>☐ Orthopedic surgeon<br>☐ Other, specify:	**1 2 3 4 5**	
1h	Optimize cardiovascular prevention: Target LDL cholesterol ≤ 2.0 g/L			**1 2 3 4**		☐ Primary care physician<br>☐ Registered nurse<br>☐ Podiatrist<br>☐ Registered nurse specialized in wounds<br>☐ Assistive Technology Service ☐Infectious disease specialist<br>☐ Vascular surgeon<br>☐ Orthopedic surgeon<br>☐ Other, specify:	**1 2 3 4 5**	
2	Role of registered nurse							
2a	Screen for peripheral artery disease: pulses and ankle-brachial index (ABI)			**1 2 3 4**		☐ Primary care physician<br>☐ Registered nurse<br>☐ Podiatrist<br>☐ Registered nurse specialized in wounds<br>☐ Assistive Technology Service ☐Infectious disease specialist<br>☐ Vascular surgeon<br>☐ Orthopedic surgeon<br>☐ Other, specify:	**1 2 3 4 5**	

2b	Screen for sensory neuropathy: 10g monofilament			1 2 3 4		☐ Primary care physician<br>☐ Registered nurse<br>☐ Podiatrist<br>☐ Registered nurse specialized in wounds<br>☐ Assistive Technology Service ☐ Infectious disease specialist<br>☐ Vascular surgeon<br>☐ Orthopedic surgeon<br>☐ Other, specify:	1 2 3 4 5	
2c	Refer to group or individual diabetes education			1 2 3 4		☐ Primary care physician<br>☐ Registered nurse<br>☐ Podiatrist<br>☐ Registered nurse specialized in wounds<br>☐ Assistive Technology Service ☐ Infectious disease specialist<br>☐ Vascular surgeon<br>☐ Orthopedic surgeon<br>☐ Other, specify:	1 2 3 4 5	
2d	Refer for nutrition assessment to evaluate nutritional status			1 2 3 4		☐ Primary care physician<br>☐ Registered nurse<br>☐ Podiatrist<br>☐ Registered nurse specialized in wounds<br>☐ Assistive Technology Service ☐ Infectious disease specialist<br>☐ Vascular surgeon<br>☐ Orthopedic surgeon<br>☐ Other, specify:	1 2 3 4 5	
2e	Evaluate the patient's support network and resources			1 2 3 4		☐ Primary care physician<br>☐ Registered nurse<br>☐ Podiatrist<br>☐ Registered nurse specialized in wounds<br>☐ Assistive Technology Service ☐ Infectious disease specialist	1 2 3 4 5	

						☐ Vascular surgeon<br>☐ Orthopedic surgeon<br>☐ Other, specify:		
2f	Identify/refer appropriately in cases of psychological distress			**1 2 3 4**		☐ Primary care physician<br>☐ Registered nurse<br>☐ Podiatrist<br>☐ Registered nurse specialized in wounds<br>☐ Assistive Technology Service ☐Infectious disease specialist<br>☐ Vascular surgeon<br>☐ Orthopedic surgeon<br>☐ Other, specify:	**1 2 3 4 5**	
3	Role of the podiatrist and/or registered nurse specialized in wounds + assistive technology services							
3a	Perform a conservative surgical debridement if indicated			**1 2 3 4**		☐ Primary care physician<br>☐ Registered nurse<br>☐ Podiatrist<br>☐ Registered nurse specialized in wounds<br>☐ Assistive Technology Service ☐Infectious disease specialist<br>☐ Vascular surgeon<br>☐ Orthopedic surgeon<br>☐ Other, specify:	**1 2 3 4 5**	
3b	Manage biofilm and investigate osteitis (bone contact and radiographs)			**1 2 3 4**		☐ Primary care physician<br>☐ Registered nurse<br>☐ Podiatrist<br>☐ Registered nurse specialized in wounds<br>☐ Assistive Technology Service ☐Infectious disease specialist<br>☐ Vascular surgeon<br>☐ Orthopedic surgeon<br>☐ Other, specify:	**1 2 3 4 5**	

3c	Take a bacterial culture if indicated			1 2 3 4		☐ Primary care physician<br>☐ Registered nurse<br>☐ Podiatrist<br>☐ Registered nurse specialized in wounds<br>☐ Assistive Technology Service ☐Infectious disease specialist<br>☐ Vascular surgeon<br>☐ Orthopedic surgeon<br>☐ Other, specify:	1 2 3 4 5	
3d	Document the wound dimensions and classify it			1 2 3 4		☐ Primary care physician<br>☐ Registered nurse<br>☐ Podiatrist<br>☐ Registered nurse specialized in wounds<br>☐ Assistive Technology Service ☐Infectious disease specialist<br>☐ Vascular surgeon<br>☐ Orthopedic surgeon<br>☐ Other, specify:	1 2 3 4 5	
3e	Evaluate the cause of the ulcer (shoes, biomechanical factors, deformities) and offload			1 2 3 4		☐ Primary care physician<br>☐ Registered nurse<br>☐ Podiatrist<br>☐ Registered nurse specialized in wounds<br>☐ Assistive Technology Service ☐Infectious disease specialist<br>☐ Vascular surgeon<br>☐ Orthopedic surgeon<br>☐ Other, specify:	1 2 3 4 5	

COMPLICATED ULCER - INITIAL PRESENTATION OR UPON REASSESSMENT (Red Section)

4	Role of the infectious disease specialist

4a	Moderate to severe cellulitis			1 2 3 4		☐ Primary care physician<br>☐ Registered nurse<br>☐ Podiatrist<br>☐ Registered nurse specialized in wounds<br>☐ Assistive Technology Service ☐Infectious disease specialist<br>☐ Vascular surgeon<br>☐ Orthopedic surgeon<br>☐ Other, specify:	1 2 3 4 5	
4b	Not responding to first-line treatment			1 2 3 4		☐ Primary care physician<br>☐ Registered nurse<br>☐ Podiatrist<br>☐ Registered nurse specialized in wounds<br>☐ Assistive Technology Service ☐Infectious disease specialist<br>☐ Vascular surgeon<br>☐ Orthopedic surgeon<br>☐ Other, specify:	1 2 3 4 5	
4c	Severity criteria: Cellulitis ≥ 2 cm, abscess, or systemic symptoms			1 2 3 4		☐ Primary care physician<br>☐ Registered nurse<br>☐ Podiatrist<br>☐ Registered nurse specialized in wounds<br>☐ Assistive Technology Service ☐Infectious disease specialist<br>☐ Vascular surgeon<br>☐ Orthopedic surgeon<br>☐ Other, specify:	1 2 3 4 5	
4d	Osteomyelitis			1 2 3 4		☐ Primary care physician<br>☐ Registered nurse<br>☐ Podiatrist<br>☐ Registered nurse specialized in wounds<br>☐ Assistive Technology Service ☐Infectious disease specialist	1 2 3 4 5	

						☐ Vascular surgeon<br>☐ Orthopedic surgeon<br>☐ Other, specify:		
4e	Bone contact or imaging evidence			**1 2 3 4**		☐ Primary care physician<br>☐ Registered nurse<br>☐ Podiatrist<br>☐ Registered nurse specialized in wounds<br>☐ Assistive Technology Service ☐ Infectious disease specialist<br>☐ Vascular surgeon<br>☐ Orthopedic surgeon<br>☐ Other, specify:	**1 2 3 4 5**	
4f	Consider intravenous antibiotic therapy			**1 2 3 4**		☐ Primary care physician<br>☐ Registered nurse<br>☐ Podiatrist<br>☐ Registered nurse specialized in wounds<br>☐ Assistive Technology Service ☐ Infectious disease specialist<br>☐ Vascular surgeon<br>☐ Orthopedic surgeon<br>☐ Other, specify:	**1 2 3 4 5**	
5	Role of the vascular surgeon							
5a	Critical ischemia			**1 2 3 4**		☐ Primary care physician<br>☐ Registered nurse<br>☐ Podiatrist<br>☐ Registered nurse specialized in wounds<br>☐ Assistive Technology Service ☐ Infectious disease specialist<br>☐ Vascular surgeon<br>☐ Orthopedic surgeon<br>☐ Other, specify:	**1 2 3 4 5**	

5b	Ankle-brachial index (ABI) ≤ 0.9 and significant alteration in arterial flow on Doppler or other assessment modality			**1 2 3 4**		☐ Primary care physician<br>☐ Registered nurse<br>☐ Podiatrist<br>☐ Registered nurse specialized in wounds<br>☐ Assistive Technology Service ☐Infectious disease specialist<br>☐ Vascular surgeon<br>☐ Orthopedic surgeon<br>☐ Other, specify:	**1 2 3 4 5**	
5c	Return to primary care for conservative treatment if non-revascularizable			**1 2 3 4**		☐ Primary care physician<br>☐ Registered nurse<br>☐ Podiatrist<br>☐ Registered nurse specialized in wounds<br>☐ Assistive Technology Service ☐Infectious disease specialist<br>☐ Vascular surgeon<br>☐ Orthopedic surgeon<br>☐ Other, specify:	**1 2 3 4**	
6	Role of the orthopedic surgeon							
6a	Charcot foot/bony spur/gangrene/deep abscess/osteomyelitis			**1 2 3 4**		☐ Primary care physician<br>☐ Registered nurse<br>☐ Podiatrist<br>☐ Registered nurse specialized in wounds<br>☐ Assistive Technology Service ☐Infectious disease specialist<br>☐ Vascular surgeon<br>☐ Orthopedic surgeon<br>☐ Other, specify:	**1 2 3 4 5**	
6b	Offloading			**1 2 3 4**		☐ Primary care physician<br>☐ Registered nurse<br>☐ Podiatrist<br>☐ Registered nurse specialized in wounds<br>☐ Assistive Technology	**1 2 3 4 5**	

						Service ☐Infectious disease specialist<br>☐ Vascular surgeon<br>☐ Orthopedic surgeon<br>☐ Other, specify:		
6c	Debridement/bone biopsy in the operating room			**1 2 3 4**		☐ Primary care physician<br>☐ Registered nurse<br>☐ Podiatrist<br>☐ Registered nurse specialized in wounds<br>☐ Assistive Technology Service ☐Infectious disease specialist<br>☐ Vascular surgeon<br>☐ Orthopedic surgeon<br>☐ Other, specify:	**1 2 3 4 5**	
	(Orange Section)							
3f	Plan dressing choices and changes, if possible, with the dedicated team			**1 2 3 4**		☐ Primary care physician<br>☐ Registered nurse<br>☐ Podiatrist<br>☐ Registered nurse specialized in wounds<br>☐ Assistive Technology Service ☐Infectious disease specialist<br>☐ Vascular surgeon<br>☐ Orthopedic surgeon<br>☐ Other, specify:	**1 2 3 4 5**	
7	CLINICAL FOLLOW-UP BY THE DEDICATED MULTIDISCIPLINARY TEAM							
7a	Dressing changes by the patient or caregiver, CLSC (Local Community Services Center), home support, or FMG nurse			**1 2 3 4**		☐ Primary care physician<br>☐ Registered nurse<br>☐ Podiatrist<br>☐ Registered nurse specialized in wounds<br>☐ Assistive Technology Service ☐Infectious disease specialist<br>☐ Vascular surgeon	**1 2 3 4 5**	

						☐ Orthopedic surgeon<br>☐ Other, specify:		
7b	TARGET ACHIEVED: ≥ 50% reduction in ulcer size after 4 weeks of treatment			1 2 3 4		☐ Primary care physician<br>☐ Registered nurse<br>☐ Podiatrist<br>☐ Registered nurse specialized in wounds<br>☐ Assistive Technology Service ☐Infectious disease specialist<br>☐ Vascular surgeon<br>☐ Orthopedic surgeon<br>☐ Other, specify:	1 2 3 4 5	
7c	TARGET NOT ACHIEVED: < 50% reduction in ulcer size after 4 weeks of treatment OR absence of granulation tissue			1 2 3 4		☐ Primary care physician<br>☐ Registered nurse<br>☐ Podiatrist<br>☐ Registered nurse specialized in wounds<br>☐ Assistive Technology Service ☐Infectious disease specialist<br>☐ Vascular surgeon<br>☐ Orthopedic surgeon<br>☐ Other, specify:	1 2 3 4 5	
7d	CONTINUE WITH CARE			1 2 3 4		☐ Primary care physician<br>☐ Registered nurse<br>☐ Podiatrist<br>☐ Registered nurse specialized in wounds<br>☐ Assistive Technology Service ☐Infectious disease specialist<br>☐ Vascular surgeon<br>☐ Orthopedic surgeon<br>☐ Other, specify:	1 2 3 4 5	

7e	REASSESS: Adherence, offloading, dressing, wound care, vascular status			1 2 3 4		☐ Primary care physician<br>☐ Registered nurse<br>☐ Podiatrist<br>☐ Registered nurse specialized in wounds<br>☐ Assistive Technology Service ☐Infectious disease specialist<br>☐ Vascular surgeon<br>☐ Orthopedic surgeon<br>☐ Other, specify:	1 2 3 4 5	
7f	CONSIDER ADVANCED THERAPIES: VAC (Vacuum-Assisted Closure), hyperbaric oxygen therapy, grafting, biological dressings			1 2 3 4		☐ Primary care physician<br>☐ Registered nurse<br>☐ Podiatrist<br>☐ Registered nurse specialized in wounds<br>☐ Assistive Technology Service ☐Infectious disease specialist<br>☐ Vascular surgeon<br>☐ Orthopedic surgeon<br>☐ Other, specify:	1 2 3 4 5	
7g	HEALING: Return to the primary care multidisciplinary team			1 2 3 4		☐ Primary care physician<br>☐ Registered nurse<br>☐ Podiatrist<br>☐ Registered nurse specialized in wounds<br>☐ Assistive Technology Service ☐Infectious disease specialist<br>☐ Vascular surgeon<br>☐ Orthopedic surgeon<br>☐ Other, specify:	1 2 3 4 5	
8	SECONDARY PREVENTION (Green Section)							
8a	Organize tailored preventive follow-up based on risk stratification			1 2 3 4		☐ Primary care physician<br>☐ Registered nurse<br>☐ Podiatrist<br>☐ Registered nurse specialized in wounds<br>☐ Assistive Technology	1 2 3 4 5	

						Service ☐Infectious disease specialist<br>☐ Vascular surgeon<br>☐ Orthopedic surgeon<br>☐ Other, specify:		
8b	Manage plantar pressures			**1 2 3 4**		☐ Primary care physician<br>☐ Registered nurse<br>☐ Podiatrist<br>☐ Registered nurse specialized in wounds<br>☐ Assistive Technology Service ☐Infectious disease specialist<br>☐ Vascular surgeon<br>☐ Orthopedic surgeon<br>☐ Other, specify:	**1 2 3 4 5**	
8c	Plan skin and nail care			**1 2 3 4**		☐ Primary care physician<br>☐ Registered nurse<br>☐ Podiatrist<br>☐ Registered nurse specialized in wounds<br>☐ Assistive Technology Service ☐Infectious disease specialist<br>☐ Vascular surgeon<br>☐ Orthopedic surgeon<br>☐ Other, specify:	**1 2 3 4 5**	
8d	Recommend regular medical follow-up			**1 2 3 4**		☐ Médecin de famille ☐☐ Primary care physician ☐ Registered nurse<br>☐ Podiatrist<br>☐ Registered nurse specialized in wounds<br>☐ Assistive Technology Service ☐Infectious disease specialist<br>☐ Vascular surgeon<br>☐ Orthopedic surgeon<br>☐ Other, specify:	**1 2 3 4 5**	

Delphi second round questionnaire (translated to English language)

Respondent characteristics

Discipline (check all that apply):
☐ Primary Care Physician
☐ Registered Nurse
☐ Registered Nurse with certification and authorized to prescribe in wound care under the Regulation on certain professional activities that can be performed by a nurse
☐ Podiatrist
☐ Registered nurse specialized in wounds
☐ Assistive Technology Service (specify profession): ________________________
☐ Physiotherapist
☐ Physiotherapist primarily working in wound care
☐ Occupational Therapist
☐ Infectious Disease Specialist
☐ Vascular Surgeon
☐ Orthopedic Surgeon
☐ Other (specify): ________________________

Sociosanitary Region of Primary Practice Setting (choose one):
☐ Bas-Saint-Laurent
☐ Saguenay – Lac-St-Jean
☐ Capitale-Nationale
☐ Mauricie et Centre-du-Québec
☐ Estrie
☐ Montreal
☐ Outaouais
☐ Abitibi-Témiscamingue
☐ Côte-Nord
☐ Nord-du-Québec
☐ Gaspésie-Îles-de-la-Madeleine
☐ Chaudières-Appalaches
☐ Laval
☐ Lanaudière
☐ Montérégie
☐ Nunavik
☐ Terres-Cries-de-la-Baie-James

Category: Clarity

42 out of the 43 items assessed were considered clear. Clarity: Easily intelligible wording, with unambiguous and unequivocal meaning.
The item "Return to primary care for conservative treatment if non-revascularizable" located in the right column (red), did not achieve consensus.

Among the following choices, which one appears the clearest to you as a replacement for this item?

1. If non-revascularizable: Schedule follow-up with vascular medicine and/or with the team/professional identified for conservative wound treatment. Consider the possibility of amputation and comfort care.
2. If non-revascularizable: Organize follow-up with the team/professional identified for conservative wound treatment. Consider the possibility of amputation and comfort

Comments:

Category: Relevance

41 out of the 43 items assessed were considered relevant. Relevance: Intervention that addresses a specific situation and meets the objectives.

Among the following choices, which one appears the most relevant to you as a replacement for the action attributed to the role of the clinical nurse "Evaluate the patient's support network and resources"?

1. Evaluate the patient's support network and resources as needed.
2. Completely remove this item from the decision support tool.

Among the following choices, which one appears the most relevant to you as a replacement for the action attributed to the role of the clinical nurse "Identify/refer appropriately in cases of psychological distress"?

1. Refer to social work or psychology as needed (would replace the items in this question and the previous one).
2. Refer to the psychosocial services at the CLSC (Local Community Services Center) as needed.
3. Completely remove this item from the decision support tool.
4. Keep the item as it is.

Comments:

Category: Feasibility

38 out of the 43 items assessed were considered feasible. Feasibility: The property of being achievable in a defined context.

Among the following choices, in the context of practice in the Quebec public healthcare network, what is the main obstacle to carrying out the action attributed to the role of the clinical nurse "Refer for nutrition assessment to evaluate nutritional status"?

1. Patient adherence to this recommendation.
2. Organizational accessibility (e.g., wait times for appointments, availability of the resource locally).
3. Material accessibility (e.g., necessary equipment for assessment not available).

4. Financial and environmental accessibility (e.g., costs for the patient, required patient travel).
5. Perceived quality of services offered (e.g., not tailored to diabetic patients with ulcers, need for healthcare professional training).

Among the following choices, in the context of practice in the Quebec public healthcare network, what is the main obstacle to carrying out the action attributed to the role of the clinical nurse "Evaluate the patient's support network and resources"?

1. Patient adherence to this recommendation.
2. Organizational accessibility (e.g., wait times for appointments, availability of the resource locally).
3. Material accessibility (e.g., necessary equipment for assessment not available).
4. Financial and environmental accessibility (e.g., costs for the patient, required patient travel).
5. Perceived quality of services offered (e.g., not tailored to diabetic patients with ulcers, need for healthcare professional training).

Among the following choices, in the context of practice in the Quebec public healthcare network, what is the main obstacle to carrying out the action attributed to the role of the clinical nurse "Identify/refer appropriately in cases of psychological distress"?

1. Patient adherence to this recommendation.
2. Organizational accessibility (e.g., wait times for appointments, availability of the resource locally).
3. Material accessibility (e.g., necessary equipment for assessment not available).
4. Financial and environmental accessibility (e.g., costs for the patient, required patient travel).
5. Perceived quality of services offered (e.g., not tailored to diabetic patients with ulcers, need for healthcare professional training).

Among the following choices, in the context of practice in the Quebec public healthcare network, what is the main obstacle to carrying out the secondary prevention action "Organize tailored preventive follow-up based on risk stratification"?

1. Patient adherence to this recommendation.
2. Organizational accessibility (e.g., wait times for appointments, availability of the resource locally).
3. Material accessibility (e.g., necessary equipment for assessment not available).
4. Financial and environmental accessibility (e.g., costs for the patient, required patient travel).
5. Perceived quality of services offered (e.g., not tailored to diabetic patients with ulcers, need for healthcare professional training).

Among the following choices, in the context of practice in the Quebec public healthcare network, what is the main obstacle to carrying out the secondary prevention action "Plan skin and nail care"?

1. Patient adherence to this recommendation.
2. Organizational accessibility (e.g., wait times for appointments, availability of the resource locally).
3. Material accessibility (e.g., necessary equipment for assessment not available).
4. Financial and environmental accessibility (e.g., costs for the patient, required patient travel).
5. Perceived quality of services offered (e.g., not tailored to diabetic patients with ulcers, need for healthcare professional training).

Comments:

Category: Responsibility

There are 16 items for which the assignment of responsibility for each action needs to be validated.

Among the following healthcare professionals, which one or ones should be responsible for the action "Early identification of the presence of an infection"?

1. Primary care physician/Nurse practitioner
2. Registered nurse
3. Primary care physician/Nurse practitioner or registered nurse

Among the following healthcare professionals, which one or ones should be responsible for the action "Optimize cardiovascular prevention: Smoking cessation"?

1. Primary care physician/Nurse practitioner
2. Registered nurse
3. Primary care physician/Nurse practitioner or registered nurse

Among the following healthcare professionals, which one or ones should be responsible for the action "Optimize cardiovascular prevention: Blood pressure ≤ 130/80 mm Hg"?

1. Primary care physician/Nurse practitioner
2. Registered nurse
3. Primary care physician/Nurse practitioner or registered nurse

Among the following healthcare professionals, which one or ones should be responsible for the action "Optimize cardiovascular prevention: Target LDL cholesterol ≤ 2.0 mmol/L"?

1. Primary care physician/Nurse practitioner
2. Registered nurse
3. Primary care physician/Nurse practitioner or registered nurse

Among the following healthcare professionals, which one or ones should be responsible for the action "Perform conservative surgical debridement if indicated"?

1. Podiatrist
2. Registered nurse specialized in wound care
3. Podiatrist or registered nurse specialized in wound care

Among the following healthcare professionals, which one or ones should be responsible for the action "Manage biofilm and investigate osteitis (bone contact and radiograph)"?

1. Podiatrist
2. Registered nurse specialized in wound care
3. Podiatrist or registered nurse specialized in wound care

Among the following healthcare professionals, which one or ones should be responsible for the action "Take a bacterial culture if indicated"?

1. Registered nurse
2. Podiatrist
3. Registered nurse specialized in wound care
4. Registered nurse or podiatrist
5. Registered nurse or registered nurse specialized in wound care
6. Podiatrist or registered nurse specialized in wound care
7. Registered nurse or podiatrist or registered nurse specialized in wound care

Among the following healthcare professionals, which one or ones should be responsible for the action "Document the wound dimensions and classify it"?

1. Registered nurse
2. Podiatrist
3. Registered nurse specialized in wound care
4. Registered nurse or podiatrist
5. Registered nurse or registered nurse specialized in wound care
6. Podiatrist or registered nurse specialized in wound care
7. Registered nurse or podiatrist or registered nurse specialized in wound care

Among the following healthcare professionals, which one or ones should be responsible for the action "Management of moderate to severe cellulitis"?

1. Primary care physician/Nurse practitioner
2. Infectious disease specialist/internal medicine specialist
3. According to the local organization of care, both choices may be suitable.

Among the following healthcare professionals, which one or ones should be responsible for the action "Charcot foot/bony spur/gangrene/deep abscess/osteomyelitis: Offloading"?

1. Podiatrist
2. Orthopedic surgeon
3. Podiatrist or orthopedic surgeon

Among the following healthcare professionals, which one or ones should be responsible for the action "Secondary prevention: Organize tailored preventive follow-up based on risk stratification"?

1. Primary care physician/Nurse practitioner
2. Registered nurse
3. Podiatrist
4. Primary care physician/Nurse practitioner or registered nurse
5. Primary care physician/Nurse practitioner or podiatrist
6. Registered nurse or podiatrist
7. Primary care physician/Nurse practitioner or registered nurse or podiatrist

Among the following healthcare professionals, which one or ones should be responsible for the action "Secondary prevention: Plan skin and nail care"?

1. Podiatrist
2. Registered nurse
3. Podiatrist or registered nurse

Among the following healthcare professionals, which one or ones should be responsible for the action "Secondary prevention: Recommend regular medical follow-up"?

1. Primary care physician/Nurse practitioner
2. Registered nurse
3. Primary care physician/Nurse practitioner or registered nurse

Comments:

Category: Additional resources

The back of the Decision Support Tool will include resources and references related to key actions in the management of diabetic foot ulcers. Please indicate the level of relevance for each proposed resource (1 = not relevant to 4 = very relevant).

Basic technique for measuring ulcers at the bedside.

1. 1 = Not relevant
2. 2 = Somewhat relevant
3. 3 = Relevant
4. 4 = Very relevant

Technique for screening neuropathy.

1. 1 = Not relevant
2. 2 = Somewhat relevant
3. 3 = Relevant
4. 4 = Very relevant

Signs and symptoms of ulcer infection.

1. 1 = Not relevant
2. 2 = Somewhat relevant
3. 3 = Relevant
4. 4 = Very relevant

Criteria for mild, moderate, or severe infection.

1. 1 = Not relevant
2. 2 = Somewhat relevant
3. 3 = Relevant
4. 4 = Very relevant

Wound culture techniques and indications.

1. 1 = Not relevant
2. 2 = Somewhat relevant
3. 3 = Relevant
4. 4 = Very relevant

Guide for the optimal use of antibiotics for diabetic foot ulcer infection.

1. 1 = Not relevant
2. 2 = Somewhat relevant
3. 3 = Relevant
4. 4 = Very relevant

Description of the ankle-brachial systolic pressure index (ABPI) measurement technique.

1. 1 = Not relevant
2. 2 = Somewhat relevant

3. 3 = Relevant
4. 4 = Very relevant

Criteria for lower limb ischemia based on different measurement techniques.

1. 1 = Not relevant
2. 2 = Somewhat relevant
3. 3 = Relevant
4. 4 = Very relevant

Classification of diabetic foot ulcers.

1. 1 = Not relevant
2. 2 = Somewhat relevant
3. 3 = Relevant
4. 4 = Very relevant

Stratification for the frequency and elements to be included in secondary prevention.

1. 1 = Not relevant
2. 2 = Somewhat relevant
3. 3 = Relevant
4. 4 = Very relevant

Recommendations for medication review at the diagnosis of a diabetic foot ulcer.

1. 1 = Not relevant
2. 2 = Somewhat relevant
3. 3 = Relevant
4. 4 = Very relevant

Description and indications for offloading modalities.

1. 1 = Not relevant
2. 2 = Somewhat relevant
3. 3 = Relevant
4. 4 = Very relevant

Guide to dressings and their indications.

1. 1 = Not relevant

2. 2 = Somewhat relevant
3. 3 = Relevant
4. 4 = Very relevant

Comments:

Delphi third round questionnaire
(translated to English language)

Respondent characteristics

Discipline (check all that apply):
 Primary Care Physician
 Registered Nurse
 Registered Nurse with certification and authorized to prescribe in wound care under the Regulation on certain professional activities that can be performed by a nurse
 Podiatrist
 Registered nurse specialized in wounds
 Assistive Technology Service (specify profession): ________________________
 Physiotherapist
 Physiotherapist primarily working in wound care
 Occupational Therapist
 Infectious Disease Specialist
 Vascular Surgeon
 Orthopedic Surgeon
 Other (specify): ________________________

Sociosanitary Region of Primary Practice Setting (choose one):
 Bas-Saint-Laurent
 Saguenay – Lac-St-Jean
 Capitale-Nationale
 Mauricie et Centre-du-Québec
 Estrie
 Montreal
 Outaouais
 Abitibi-Témiscamingue
 Côte-Nord
 Nord-du-Québec
 Gaspésie-Îles-de-la-Madeleine
 Chaudières-Appalaches
 Laval
 Lanaudière
 Montérégie
 Nunavik
 Terres-Cries-de-la-Baie-James

Category: Clarity

Clarity: wording easily understandable, with a clear and unambiguous meaning.

The item "Return to first-line for conservative treatment if non-revascularizable" located in the right column (red) did not reach a consensus.

Among the following choices, which one appears to you as the clearest to replace this item?

1. If non-revascularizable: Plan follow-up with vascular medicine and/or the team/professional identified for conservative wound treatment. Consider the possibility of amputation and comfort care.
2. If non-revascularizable: Organize follow-up with the team/professional identified for conservative wound treatment. Consider the possibility of amputation and comfort care.

Category: Relevance

Relevance: an intervention that addresses a specific situation and meets the objectives.

Among the following choices, which one do you find most relevant to replace the action attributed to the role of the clinical nurse "Assess the patient's support network and resources"?

1. Assess the patient's support network and resources as needed.
2. Completely remove this item from the decision support tool.

Category: Feasibility

Feasibility: the property of being achievable in a defined context.

In a context of practice in the Quebec public healthcare network, what is the main obstacle to carrying out the secondary prevention action "Plan skin and nail care"?

1. Organizational accessibility (e.g., waiting times for care, availability of local resources)
2. Perceived quality of services offered (e.g., not tailored to diabetic patients with ulcers, need for healthcare professional training)

Category: Responsibility

Responsibility: Assignment of responsibility to the appropriate healthcare provider.

Among the following choices, which healthcare professionals should be responsible for the “Management of moderate to severe cellulitis”?

1. Primary care physician/Nurse practitioner
2. Infectious disease specialist/internal medicine specialist
3. According to the local organization of care, both choices may be suitable
