# Supplementary material for: Developing an interprofessional decision support tool for diabetic foot ulcers management in primary care within the family medicine group model: a Delphi study in Canada
Source: BMC Prim Care. 2024 Apr 20;25:123. doi: 10.1186/s12875-024-02387-4 (PMC11031884; doi:10.1186/s12875-024-02387-4)
Supplement: Supplementary file 2 — Additional file 2. Final version of the decision support tool in colors translated to English. [file 12875_2024_2387_MOESM2_ESM.pdf]

DECISION SUPPORT TOOL FOR ASSESSMENT AND MANAGEMENT OF A PERSON WITH A DIABETIC FOOT ULCER

UQTR Université du Québec à Trois-Rivières · Groupe de Médecine de Famille universitaire de Trois-Rivières · Université de Montréal · Centre intégré universitaire de santé et de services sociaux de la Mauricie-et-du-Centre-du-Québec, Québec

Uncomplicated ulcer

Initial presentation in primary care

Role of the primary care physician/nurse practitioner [1-3]

- Initiating oral antibiotic therapy for mild infections [2a, 3b, 4-6]
- Managing pain [1a, 2b, 3, 5]
- Controlling blood glucose(HbA1c ≤ 7.0% [53 mmol/mol] or individualized target) [2]
- Reviewing medication in collaboration with the pharmacist [1-3, 5]
- Early Identification of the presence of an infection [2a, 3b, 4] ○
- Optimizing cardiovascular prevention [2]:
  - Smoking cessation
  - Blood pressure ≤ 130/80 mm Hg
  - LDL cholesterol ≤ 2.0 mmol/L

Role of the registered nurse (RN) [1-3]

- Screening for peripheral arterial disease: pulse and ABI [1, 2b, 3a]
- Screening for sensory neuropathy: 10 g monofilament [1a, 2b, 3a, 5, 6]
- Referring for group or individual diabetes education [1, 3]
- Referring for assessment of nutritional status [1, 2c, 3]
- Assessing the patient's support network and resources [1a, 2, 5, 7]
- Identifying/referring appropriately for psychological distress [1a, 2a, 3, 5-7]

Role of the podiatrist [1a, 3, 5, 6], registered nurse (RN) specialized in wound and/or stomatologist [1a, 3, 7] + technical aids service [1, 3]

- Performing conservative surgical debridement if safe [1b, 3, 5-7]
- Managing biofilm [1a, 3b, 4, 7] and looking for osteitis (bone contact □ and x-rays) [1a, 2a, 3b, 4, 7]
- Collecting for bacteriological culture if indicated [3b, 4]
- Documenting wound [1a, 7] dimensionsand classifying [1b, 2a, 3, 5, 6]
- Assessing the cause of the ulcer (footwear, biomechanical cause, ✤ deformities) [1ab, 3, 5-7] and offloading [1b, 2a, 3d, 5, 6]

Secondary prevention

- Organizing a preventive follow-up adapted to the risk (stratification) [1b, 3, 6]
- Managing plantar pressures [1b, 2a, 3d, 5, 6]
- Planning skin and nail care [1b, 2a, 3, 5, 6]
- Recommending regular medical follow-up [2, 5]

Clinical follow-up by the dedicated interdisciplinary team or identified professional [1ab, 2a, 3d, 5-7]

in collaboration with the patient and caregivers, CLSC, home support and primary care medical team

Dressing changes, debridement [1ab, 7], offloading [1b, 2a, 3d, 5, 6], and periodic wound reassessment [1-7]

Target reached [3, 7]

≥ 50% reduction in ulcer size after 4 weeks of treatment

Continuing wound care

Healing

Return to the multidisciplinary primary care team

Reviewing treatment objective

Non-healing or non-healable

Target not reached [3, 7]

< 50% reduction in ulcer size after 4 weeks of treatment OR absence of granulation tissue

Reassessing [1ab, 2a, 3, 7]

- Adherence
- Offloading
- Dressing
- Wound care
- Vascular condition

Considering advanced therapies [1, 3, 7]

- Negative pressure therapy
- Hyperbaric treatment
- Grafting
- Biological dressings

Complicated ulcer

Initial presentation OR at the time of revaluation

Vascular Surgeon [1b, 3a, 5, 6]

Critical Limb Ischemia [1b, 3a, 5, 6] △

ABI ≤ 0.9 and significant hemodynamic restriction according to a Doppler or another assessment modality as locally available

If non-revascularizable: organize follow-up by identified team/professional for conservative wound management. Consider amputation and palliative care.

Infectiologist/internist [1b, 2a, 3b, 4-6]

Consider intravenous antibiotic therapy

Moderate to severe cellulitis ○

Not responding to primary care therapy

or

With severity criteria: cellulitis ≥ 2 cm, abscess or systemic symptoms [1b, 3b, 4]

Osteitis □

Bone contact or evidence of osteitis on imaging [1b, 3b, 4, 5]

Orthopedist/Podiatrist [1b, 3bd, 4, 5]

Charcot foot / bone spur / gangrene / deep abscess / osteitis ✤

For surgical offloading and debridement, bone biopsy in the operating room, minor/major amputation

Key

○ △ □ ✤ : same symbols for associated actions

ABI: Ankle-brachial systolic pressure index

ADDITIONAL TOOLS AND RESOURCES

Measuring the ulcer

Wounds UK

Screening for peripheral neuropathy

Diabetes Canada

Interpreting the vascular assessment of the lower limb

Wounds Canada (p. 14)

Detecting signs and symptoms of wound infection

IWGDF (p. 14)

INESSS

IWII (p. 9)

Diabetes Canada (Table 3)

Classifying diabetic ulcers

IWGDF (SINBAD System, Table 2, p. 14)

Providing descriptions and indications of offloading modalities

IWGDF (p. 4-5)

Determining risk stratification and frequency of follow-up

Wounds Canada

Key

INESSS: Institut national d'excellence en santé et services sociaux (National institute of excellence in health and social services)
IWGDF: International Working Group on the Diabetic Foot
IWII: International Wound Infection Institute

References

1. Wound Canada. Foundations of Best Practice for Skin and Wound Management. In: Best Practice Recommendations 2017 Update 2017 [Available from: https://www.woundscanada.ca/health-care-professional/publications/dfc-2.]
   a. Botros M, Kuhnke J, Embil J, Goettl K, Morin C, Parsons L, et al. Best practice recommendations for the prevention and management of diabetic foot ulcers In: Foundations of Best Practice for Skin and Wound Management. A supplement of Wound Care Canada2017. [Available from: www.woundscanada.ca/docman/public/health-care-professional/bpr-workshop/895-wc-bpr-prevention-andmanagement-of-diabetic-foot-ulcers-1573r1e-final/file]
   b. Orsted H, Keast D, Forest-Lalande L, Kuhnke J, O'Sullivan-Drombolis D, Jin S. Best practice recommendations for the prevention and management of wounds. In: Foundations of Best Practice for Skin and Wound Management. A supplement of Wound Care Canada 2017 [Available from: www.woundscanada.ca/docman/public/health-care-professional/bpr-workshop/165-wc-bpr-prevention-andmanagement-of-wounds/file.]
2. Diabetes Canada Clinical Practice Guidelines Expert Committee. Diabetes Canada 2018 Clinical Practice Guidelines for the Prevention and Management of Diabetes in Canada. Can. 2018;42:S1-S325
   a. Embil JM, Albalawi Z, Bowering K, Trepman E. 2018 Clinical Practice Guidelines: Foot Care. Can. 2018;42:S222-S227
   b. Bril V, Breiner A, Perkins BA, Zochodne D, Committee DCCPGE. Neuropathy. Can. 2018;42:S217-S221
   c. Sievenpiper JL, Chan CB, Dworatzek PD, Freeze C, Williams SL, Committee DCCPGE. Nutrition therapy. Can. 2018;42:S64-S79
3. Bus S, Monteiro-Soares M, Game F, van Netten J, Apelqvist J, Fitridge R, et al. 2023 IWGDF Guidelines on the Prevention and Management of Diabetic Foot Disease 2023 [Available from: https://iwgdfguidelines.org/guidelines-2023/all-guidelines-2023.]
   a. Hinchliffe RJ, Forsythe RO, Apelqvist J, Boyko EJ, Fitridge R, Hong JP, et al. Guidelines on diagnosis, prognosis, and management of peripheral artery disease in patients with foot ulcers and diabetes (IWGDF 2019 update). Diabetes Metab Res Rev. 2020;36:e3276
   b. Senneville E, Albalawi Z, van Asten S, Abbas Z, Allison G, Aragón-Sánchez J, et al. Guidelines on the diagnosis and treatment of foot infection in persons with diabetes IWGDF/IDSA 2023 2023 [Available from: https://iwgdfguidelines.org/wp-content/uploads/2023/05/IWGDF-2023-04-Infection-Guideline.pdf.]
   c. Wukich D, Schaper N, Gooday C, Bal A, Bem R, Chhabra A, et al. Guidelines on the diagnosis and treatment of active Charcot neuroosteoarthropathy in persons with diabetes mellitus: The International Working Group on the Diabetic; 2023 [Part of the 2023 IWGDF Guidelines on the prevention and management of diabetes-related foot disease]. [Available from: https://iwgdfguidelines.org/charcot-2023/.]
   d. Bus S, Armstrong D, Crews R, Gooday C, Jarl G, Kirketerp-Møller K, et al. Guidelines on offloading foot ulcers in persons with diabetes Part of the 2023 IWGDF Guidelines on the prevention and management of diabetes-related foot disease: International Working Group on the Diabetic Foot; 2023 [Available from: https://iwgdfguidelines.org/offloading-guideline-2023/.]
4. Swanson T, Ousey K, Haesler E, Bjarnsholt T, Carville K, Idensohn P, et al. IWII Wound Infection in Clinical Practice consensus document: 2022 update. J Wound Care. 2022;31(Sup12):S10-S21
5. National Institute for Health and Care Excellence. Diabetic foot problems: prevention and management [NG19] 2015 [Available from: https://www.nice.org.uk/guidance/ng19.]
6. Kaminski M, Golledge J, Lasschuit J, Schott K, Charles J, Cheney J. Australian Diabetes-Related Foot Disease Guidelines & Pathway Project 2022 [Available from: https://diabetesfeetaustralia.stonly.com/kb/en.]
7. Sibbald RG, Elliott JA, Persaud-Jaimangal R, Goodman L, Armstrong DG, Harley C, et al. Wound bed preparation 2021. Wound Healing Southern Africa. 2021;14(2):52-62
