# Supplementary material for: Developing an interprofessional decision support tool for diabetic foot ulcers management in primary care within the family medicine group model: a Delphi study in Canada
Source: BMC Prim Care. 2024 Apr 20;25:123. doi: 10.1186/s12875-024-02387-4 (PMC11031884; doi:10.1186/s12875-024-02387-4)
Supplement: Supplementary file 3 — Additional file 3. Original French version of the final version of the decision support tool. [file 12875_2024_2387_MOESM3_ESM.pdf]

OUTIL D'AIDE À LA DÉCISION POUR L'ÉVALUATION ET LA PRISE EN CHARGE D'UNE PERSONNE DIABÉTIQUE AVEC ULCÈRE AU PIED

UQTR Université du Québec à Trois-Rivières
Groupe de médecine de famille universitaire de Trois-Rivières
Université de Montréal
Centre intégré universitaire de santé et de services sociaux de la Mauricie-et-du-Centre-du-Québec
Québec

Ulcère non compliqué

Présentation initiale en 1re ligne

Rôle du médecin de famille/IPSPL [1-3]

- Initier une antibiothérapie orale en présence d'une infection légère [2a, 3b, 4-6]
- Gérer la douleur [1a, 2b, 3, 5]
- Contrôler la glycémie (HbA_{1c} ≤ 7,0% ou cible individualisée) [2]
- Réviser la médication en collaboration avec le pharmacien [1-3, 5]
- Identifier précocément la présence d'une infection [2a, 3b, 4]
- Optimiser la prévention cardiovasculaire [2]:
  - Arrêt tabagique
  - Pression artérielle ≤ 130/80 mm Hg
  - Cholestérol LDL ≤ 2,0 mmol/L

Rôle de l'infirmier.ère clinicien.ne [1-3]

- Dépister la maladie artérielle périphérique: pouls et IPSCB [1, 2b, 3a]
- Dépister la neuropathie sensitive: monofilament 10 g [1a, 2b, 3a, 5, 6]
- Référer pour enseignement sur le diabète de groupe ou individuel [1, 3]
- Référer en nutrition pour une évaluation du statut nutritionnel [1, 2c, 3]
- Évaluer le réseau de soutien et les ressources du patient [1a, 2, 5, 7]
- Identifier/référer de façon appropriée en cas de souffrance psychologique [1a, 2a, 3, 5-7]

Rôle du podiatre [1a, 3, 5, 6] infirmier.ère compétent.e en soins de plaies et/ou stomothérapeute [1a, 3, 7] + service d'aides techniques [1, 3]

- Effectuer un débridement chirurgical conservateur si sécuritaire [1b, 3, 5-7]
- Prendre en charge le biofilm [1a, 3b, 4, 7] et rechercher une ostéite (contact osseux et radiographies) [1a, 2a, 3b, 4, 7]
- Prélever pour une culture bactériologique si indiqué [3b, 4]
- Documenter les dimensions de la plaie [1a, 7] et classifier [1b, 2a, 3, 5, 6]
- Évaluer la cause de l'ulcère (chaussures, cause biomécanique, déformations) [1ab, 3, 5-7] et mettre en décharge [1b, 2a, 3d, 5, 6]

Prévention secondaire

- Organiser un suivi préventif adapté au risque (stratification) [1b, 3, 6]
- Gérer les pressions plantaires [1b, 2a, 3d, 5, 6]
- Planifier les soins de la peau et des ongles [1b, 2a, 3, 5, 6]
- Recommander un suivi médical régulier [2, 5]

Suivi clinique par l'équipe interdisciplinaire dédiée ou le professionnel identifié dans son milieu [1ab, 2a, 3d, 5-7]

en collaboration avec le patient et les proches aidants, le CLSC, le soutien à domicile et l'équipe médicale de 1re ligne

Changement de pansements, débridements [1ab, 7], décharge [1b, 2a, 3d, 5, 6] et réévaluation périodique de la plaie [1-7]

Cible atteinte [3, 7]

≥ 50 % de réduction de la taille de l'ulcère après 4 semaines de traitement

Poursuivre les soins

Guérison

Retourner à l'équipe multidisciplinaire de 1re ligne

Réviser l'objectif de soin

Maintien ou incurable

Cible non atteinte [3, 7]

< 50 % de réduction de la taille de l'ulcère après 4 semaines de traitement OU absence de tissu de granulation

Réévaluer [1ab, 2a, 3, 7]

- Adhésion
- Décharge
- Pansement
- Soins de plaie
- État vasculaire

Considérer les thérapies avancées [1, 3, 7]

- Thérapie par pression négative
- Hyperbare
- Greffe
- Pansements biologiques

Ulcère compliqué

Présentation initiale OU lors de la réévaluation

Chirurgien vasculaire [1b, 3a, 5, 6]

Ischémie critique [1b, 3a, 5, 6]

IPSCB ≤ 0,9 et frein hémodynamique significatif au Doppler ou autre modalité d'évaluation selon disponibilité locale

Si non revascularisable: organiser suivi par équipe/professionnel identifié pour traitement conservateur de la plaie. Considérer possibilité d'amputation et soins de confort.

Infectiologue/interniste [1b, 2a, 3b, 4-6]

Considérer antibiothérapie intraveineuse

Cellulite modérée à sévère

Ne répondant pas au traitement de 1re ligne
ou
Avec critères de sévérité: cellulite ≥ 2 cm, abcès ou symptômes systémiques [1b, 3b, 4]

Ostéite

Contact osseux ou évidence d'ostéite à l'imagerie [1b, 3b, 4, 5]

Orthopédiste/podiatre [1b, 3bd, 4, 5]

Pied de Charcot / éperon osseux / gangrène / abcès profond / ostéite

Pour décharge et débridement chirurgicaux, biopsie osseuse en salle d'opération, amputation mineure/majeure

Légende

: actions associées
IPSCB : indice de pression systolique cheville-bras
IPSPL : infirmière praticienne spécialisée en 1re ligne

OUTILS ET RESSOURCES SUPPLÉMENTAIRES

Prise de mesure de l'ulcère

RQSP

Dépistage de la neuropathie périphérique

Diabète Canada

Interprétation de l'évaluation vasculaire du membre inférieur

INESSS (p. 4)

Signes et symptômes de l'infection de plaie

INESSS (p. 11)

INESSS (antibiotiques)

IWII (p. 9)

Diabète Canada (Tableau 3, p. S224)

Classification des ulcères diabétiques

IWGDF (Système SINBAD, Table 2, p. 14)

Descriptions et indications des modalités de décharge

IWGDF (p. 4-5)

OIIQ (Tableau 4, p. 42)

Stratification du risque et fréquence du suivi

Plaies Canada

Légende :

INESSS : Institut national d'excellence en santé et services sociaux
IWGDF : International Working Group on the Diabetic Foot
IWII : International Wound Infection Institute
OIIQ : Ordre des infirmières et infirmiers du Québec
RQSP : Regroupement québécois en soins de plaies

References

1. Wound Canada. Foundations of Best Practice for Skin and Wound Management. In: Best Practice Recommendations 2017 Update 2017 [Available from: https://www.woundscanada.ca/health-care-professional/publications/dfc-2.]
   a. Botros M, Kuhnke J, Embil J, Goettl K, Morin C, Parsons L, et al. Best practice recommendations for the prevention and management of diabetic foot ulcers In: Foundations of Best Practice for Skin and Wound Management. A supplement of Wound Care Canada2017. [Available from: www.woundscanada.ca/docman/public/health-care-professional/bpr-workshop/895-wc-bpr-prevention-andmanagement-of-diabetic-foot-ulcers-1573r1e-final/file]
   b. Orsted H, Keast D, Forest-Lalande L, Kuhnke J, O'Sullivan-Drombolis D, Jin S. Best practice recommendations for the prevention and management of wounds. In: Foundations of Best Practice for Skin and Wound Management. A supplement of Wound Care Canada 2017 [Available from: www.woundscanada.ca/docman/public/health-care-professional/bpr-workshop/165-wc-bpr-prevention-andmanagement-of-wounds/file.]
2. Diabetes Canada Clinical Practice Guidelines Expert Committee. Diabetes Canada 2018 Clinical Practice Guidelines for the Prevention and Management of Diabetes in Canada. Can. 2018;42:S1-S325
   a. Embil JM, Albalawi Z, Bowering K, Trepman E. 2018 Clinical Practice Guidelines: Foot Care. Can. 2018;42:S222-S227
   b. Bril V, Breiner A, Perkins BA, Zochodne D, Committee DCCPGE. Neuropathy. Can. 2018;42:S217-S221
   c. Sievenpiper JL, Chan CB, Dworatzek PD, Freeze C, Williams SL, Committee DCCPGE. Nutrition therapy. Can. 2018;42:S64-S79
3. Bus S, Monteiro-Soares M, Game F, van Netten J, Apelqvist J, Fitridge R, et al. 2023 IWGDF Guidelines on the Prevention and Management of Diabetic Foot Disease 2023 [Available from: https://iwgdfguidelines.org/guidelines-2023/all-guidelines-2023.]
   a. Hinchliffe RJ, Forsythe RO, Apelqvist J, Boyko EJ, Fitridge R, Hong JP, et al. Guidelines on diagnosis, prognosis, and management of peripheral artery disease in patients with foot ulcers and diabetes (IWGDF 2019 update). Diabetes Metab Res Rev. 2020;36:e3276
   b. Senneville E, Albalawi Z, van Asten S, Abbas Z, Allison G, Aragón-Sánchez J, et al. Guidelines on the diagnosis and treatment of foot infection in persons with diabetes IWGDF/IDSA 2023 2023 [Available from: https://iwgdfguidelines.org/wp-content/uploads/2023/05/IWGDF-2023-04-Infection-Guideline.pdf.]
   c. Wukich D, Schaper N, Gooday C, Bal A, Bem R, Chhabra A, et al. Guidelines on the diagnosis and treatment of active Charcot neuroosteoarthropathy in persons with diabetes mellitus: The International Working Group on the Diabetic; 2023 [Part of the 2023 IWGDF Guidelines on the prevention and management of diabetes-related foot disease]. [Available from: https://iwgdfguidelines.org/charcot-2023/.]
   d. Bus S, Armstrong D, Crews R, Gooday C, Jarl G, Kirketerp-Møller K, et al. Guidelines on offloading foot ulcers in persons with diabetes Part of the 2023 IWGDF Guidelines on the prevention and management of diabetes-related foot disease: International Working Group on the Diabetic Foot; 2023 [Available from: https://iwgdfguidelines.org/offloading-guideline-2023/.]
4. Swanson T, Ousey K, Haesler E, Bjarnsholt T, Carville K, Idensohn P, et al. IWII Wound Infection in Clinical Practice consensus document: 2022 update. J Wound Care. 2022;31(Sup12):S10-S21
5. National Institute for Health and Care Excellence. Diabetic foot problems: prevention and management [NG19] 2015 [Available from: https://www.nice.org.uk/guidance/ng19.]
6. Kaminski M, Golledge J, Lasschuit J, Schott K, Charles J, Cheney J. Australian Diabetes-Related Foot Disease Guidelines & Pathway Project 2022 [Available from: https://diabetesfeetaustralia.stonly.com/kb/en.]
7. Sibbald RG, Elliott JA, Persaud-Jaimangal R, Goodman L, Armstrong DG, Harley C, et al. Wound bed preparation 2021. Wound Healing Southern Africa. 2021;14(2):52-62
